# Transmission investigation of *Mycoplasma synoviae* in Chinese indigenous chickens

**DOI:** 10.3389/fvets.2025.1555604

**Published:** 2025-05-13

**Authors:** Liping Yin, Yan Luo, Changming Li, Hanjie Yin, Qiang Zhou, Shutao Cui, Moru Xu, Haitao Zhang, Aijian Qin, Li Wang

**Affiliations:** ^1^Jiangsu Lihua Animal Husbandry Co., Ltd., Changzhou, Jiangsu, China; ^2^Ministry of Education Key Lab for Avian Preventive Medicine, College of Veterinary Medicine, Yangzhou University, Yangzhou, Jiangsu, China; ^3^Jiangsu Co-innovation Center for Prevention and Control of Important Animal Infectious Diseases and Zoonoses, College of Veterinary Medicine, Yangzhou University, Yangzhou, Jiangsu, China

**Keywords:** Chinese indigenous chickens, *Mycoplasma synoviae*, vertical transmission, egg production, horizontal transmission

## Abstract

*Mycoplasma synoviae* (MS) induces avian synovitis, presenting with tendon inflammation, and respiratory distress, ultimately compromising poultry health and farm productivity. To investigate the epidemiological characteristics of MS in Chinese indigenous chickens, a comprehensive study was conducted on chicken flocks from three breeder farms in Jiangsu, China. A total of 113 batches of chicken flocks were screened using real-time polymerase chain reaction (qPCR). Among 3,284 choanal cleft swab samples collected from chickens aged 2 to over 25 weeks, 1,695 tested positive for MS. Notably, the MS-positive rate increased significantly in chickens aged 8 to 25 weeks. Interestingly, none of the chicken embryo samples (0/322) and only two one-day-old chickens (2/927) tested positive for MS infection. In contrast, *Mycoplasma gallisepticum* (MG) infection was more prevalent, particularly in unhatched embryos (158/294), primarily due to air sac contamination. All offspring from MS-positive parent flocks aged 27 to 38 weeks tested negative for MS. To further explore the influence of the breeding environment, chickens from the same batch were raised either in breeder farms or isolators for 17 weeks. Chickens housed in breeder farms exhibited MS nucleic acid and antibody positivity from 9 to 17 weeks, whereas those raised in isolators remained MS-free throughout the study. These findings indicate that vertical transmission of MS in Chinese indigenous chickens is rare, with horizontal transmission being the predominant mode of spread.

## 1 Introduction

The genus *Mycoplasma* includes 25 species associated with poultry, among which *Mycoplasma synoviae* (MS) and *Mycoplasma gallisepticum* (MG) are major pathogens in chickens globally. MS causes inflammation in the synovial membranes of joints and tendon sheaths, swelling of tarsal joints and paws, even paralysis. Reduced egg production, subclinical upper respiratory infection and retarded growth is also obvious in MS-affected chicken. MG is responsible for chronic respiratory diseases including respiratory rales, coughing, nasal discharge, resulting in reduced growth performance, feed conversion efficiency, and carcass quality (1–3). Infection of either MS or MG causes higher mortality rates, increased vaccination and treatment costs, and susceptibility to secondary bacterial or viral infections, leading to significant economic losses in the poultry industry (4–6).

MS can be transmitted both vertically and horizontally (7). Vertical transmission occurs primarily through infected embryos and is particularly severe during the first 4 to 6 weeks of egg production (8). Horizontal transmission takes place through airborne aerosols and contaminated dust (1, 9, 10). Preventive measures for MS infection include biosecurity management, drug treatment, and vaccination (11). Biosecurity management focuses on sourcing breeding stock from MS-free farms and maintaining uninfected flocks. Antibiotics, such as tetracyclines, macrolides, tiamulin, and lincomycin, can be used for prevention and treatment (12, 13). Currently available vaccines include attenuated live vaccines [F (FVax-MGH), 6/85 (Mycovac-LH), Ts-11 (MG ts-11H), and MS-H (Vassafe MS)], which are typically administered via nasal or eye drops. Inactivated and subunit vaccines are also commercially available (14–16) and had made great contributions to the control of MS.

Since 2010, cases of MS infection have been reported in indigenous chickens in China (5, 6, 17), with an increasingly significant impact on poultry production in recent years. This study investigates the vertical and horizontal transmission dynamics of MS in three indigenous chicken breeding farms in Jiangsu Province, China. The findings aim to provide valuable insights for the comprehensive prevention and control of MS infections in Chinese indigenous chickens.

## 2 Materials and methods

### 2.1 Background of chicken flocks

**Figure 1:**
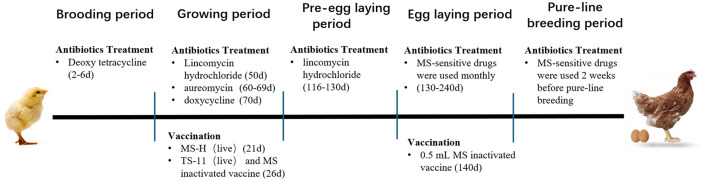
Antibiotic used and immunization protocol for chicken farms.

Three Chinese indigenous chicken breeding farms in Jiangsu Province were included in this study. Information about antibiotics and vaccines applied in the farms is shown in Figure 1.

### 2.2 Clinical samples collection

To evaluate the MS infection status in breeding chickens, a total of 3,284 choanal cleft swab samples were collected from 113 chicken flocks across three indigenous chicken breeding farms. The farms were situated 2 to 6 km apart, with no slaughterhouses, live poultry markets, or other potential pathogen transmission sources located within a 5 km radius. The sampled flocks ranged in age from 2 to 64 weeks, with ~40 choanal cleft swab samples collected per flock.

To further assess MS infection in chicken embryos, 322 chicken embryo samples (egg yolk sacs and allantoic fluid were mixed) were collected from nine different layer breeds. Additionally, tracheal and lung samples were obtained from 927 one-day-old chickens to monitor MS and MG infection status.

### 2.3 DNA/RNA extraction and qPCR detection

All collected samples were placed in EP tubes containing 0.5 mL of PBS and grinded. The sample supernatant was collected after centrifugation at 5,000 rpm for 5 min. A volume of 200 μL of the supernatant was added to the sample well of the DNA/RNA extraction kit (Jifan Biology Co., LTD, Changzhou, China), and the total DNA was extracted using an automatic nucleic acid extractor (Xinchun Biology Co., LTD, Changzhou, China).

Primers were synthesized according to the previous report (10) (Supplementary Table S1). The qPCR procedure included an initial denaturation cycle of 3 min at 95°C, followed by 40 cycles of denaturation for 30 s at 95°C, annealing and extension at 60°C and for 60 s. The fluorescence signal was collected with the LightCycler 96 Quantitative fluorescence PCR instrument (Roche Pharmaceutical Co., LTD.).

### 2.4 Detection of the sera antibodies

Serological analysis was performed using an ELISA commercial test (BioCheck MS ELISA kit; BioCheck, ER, Reeuwijk, Netherlands). Following the instructions, the S/P ratio of the sample was calculated with the optical density (OD) at 450 nm. Sera samples with an S/P ratio ≥0.5 were regarded as positive.

### 2.5 Detection of vertical transmission of MS

To investigate the potential for vertical transmission of *Mycoplasma synoviae* (MS) in Chinese indigenous chickens, offspring were derived from MS-positive roosters and hens (5 roosters and 20 hens). Semen was collected, pooled, and used for artificial insemination of the hens twice weekly. From 27 to 38 weeks of age, choanal cleft swabs were collected weekly from both male and female chickens and tested for MS using qPCR. Eggs produced by the hens were incubated and tracheal and lung tissues from 1-day-old offspring were analyzed for MS infection via qPCR.

### 2.6 Detection of horizon transmission of MS

To evaluate the influence of the breeding environment on MS infection status, 60 one-day-old chickens from farm A (which had a high MS-positive rate) were randomly divided into two groups, with 30 chickens per group. Chickens in group 1 were raised in three isolators, while those in group 2 were housed in three breeding farms. All other rearing conditions were maintained consistently. Choanal cleft swabs and serum samples were collected from chickens in both groups at 1, 5, 7, 9, 12, and 17 weeks of age for analysis.

### 2.7 MLST typing

Isolation and identification of MS were performed on the chickens suspected of MS infection in the breeding farm, and DNA was extracted from the purified strains. Seven housekeeping genes (*adk, atpG, efp, gmk, nagC, ppa*, and *recA)* were used for MLST typing. Corresponding primers were obtained from the PubMLST website (https://pubmlst.org/organisms/mycoplasma-synoviae). Following amplification, the products were sequenced by Shanghai Biotechnology Co., Ltd. The DNA sequences of the seven housekeeping genes were aligned and assembled using Lasergene 7 software. The sequence type (ST) was determined based on the allele pattern identified.

## 3 Results

### 3.1 MS infection status in different breeding farms

A total of 3,284 anal swab samples were collected from chickens aged 2 to 60 weeks across three farms and tested for MS using qPCR. Results are summarized in Table 1. During the 2–7 week period, MS-positive rates were low, with only 1 out of 140 chickens testing positive on farm B. However, between 8 and 12 weeks, MS infection rates increased significantly across all farms. More than half of the flocks tested positive during this period. Specifically, farm A exhibited a 100% group positivity rate, with an individual positivity rate of 81.7%. Farms B and C showed individual positivity rates of 44.2% and 38.8%, respectively. From 13 to 25 weeks, MS-positive rates gradually declined on farms A and B but increased on farm C. After 25 weeks, all three farms reached a 100% group positivity rate, with individual positivity rates of 71.5%, 96.3%, and 74.2% for farms A, B, and C, respectively.

**Table 1:** MS infection in chicken flocks at different ages from different farms.

**Farm**	**Positive rate**	**2–7 weeks old**	**8–12 weeks old**	**13–24 weeks old**	**Above 25 weeks old**
Farm A	The proportion of MS positive groups	0.0% (0/4)	100.0% (4/4)	100.0% (11/11)	100.0% (23/23)
	Total MS positive rates	0.0% (0/80)	81.7% (98/120)	60.9% (262/430)	71.5% (388/534)
Farm B	The proportion of MS positive groups	14.4% (1/7)	75.0% (3/4)	85.7% (12/14)	100.0% (9/9)
	Total MS positive rates	0.7% (1/140)	44.2% (53/120)	37.4% (142/380)	96.3% (154/160)
Farm C	The proportion of MS positive groups	0.0% (0/4)	57.1% (4/7)	92.3% (12/13)	100.0% (13/13)
	Total MS positive rates	0.0% (0/100)	38.9% (109/280)	47.0% (296/630)	74.2% (193/260)

In summary, chickens showed no MS infection at an early age, but the positive rates increased sharply at 8 weeks and continued to rise steadily thereafter.

### 3.2 MS and MG infection in chicken embryos samples and 1-day-old chickens

A total of 322 chicken embryo samples from two breeds were collected and tested for *Mycoplasma synoviae* (MS) infection using quantitative PCR (qPCR), with all samples testing negative (data not shown). Additionally, 927 one-day-old chickens from Breed 1 and Breed 2 were screened for MS infection (Table 2). Only two unhatched chickens from Breed 1 tested positive for MS.

**Table 2:** Results of *Mycoplasma* positive rate in 1-day-old chicken samples at different ages.

**Breed**	**Chicken status**	**Sample numbers**	**MG positive rate**	**MS positive rate**	**Air sac contamination ratio**
Breed 1	Healthy	123	0.0% (0/123)	0.0% (0/123)	0.0% (0/123)
	Weak	141	6.7% (8/141)	0.0% (0/141)	2.8% (4/141)
	Pipped but unhatched	106	86.8% (92/106)	1.9% (2/106)	50.0% (53/106)
Breed 2	Healthy	185	0.0% (0/185)	0.0% (0/185)	0.0% (0/185)
	Weak	184	1.1% (2/184)	0.0% (0/184)	0.0% (0/184)
	Pipped but unhatched	188	35.1% (66/188)	0.0% (0/188)	17.0% (32/188)

**Figure 2:**
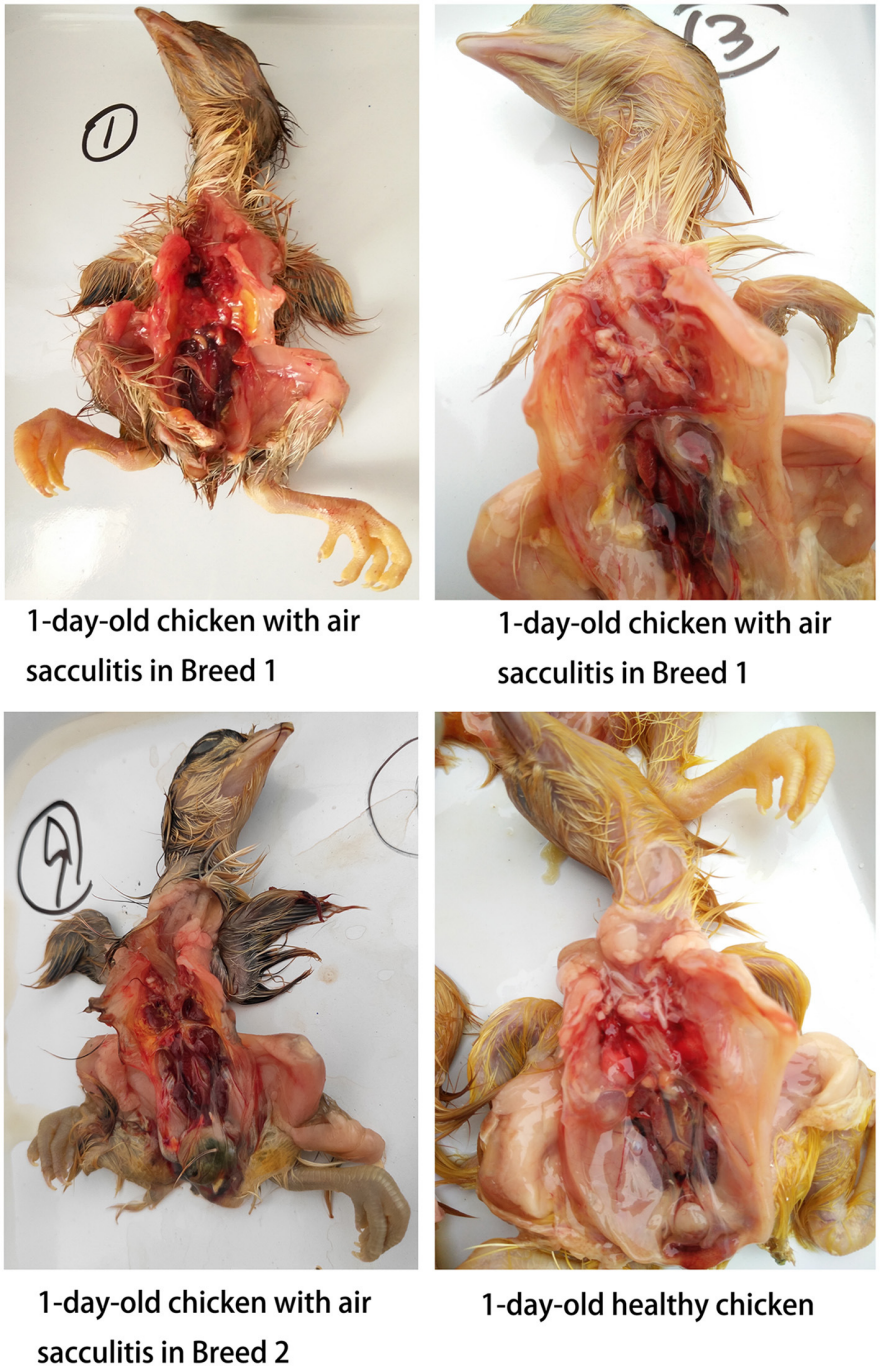
Chicken with air sacculitis from different breeds.

In contrast, *Mycoplasma gallisepticum* (MG) infection was more prevalent, particularly in unhatched chickens. All healthy chickens were MG-negative, while 10 out of 325 weak chickens tested positive, including 4 with air sac contamination. Notably, 53.7% (158/294) of unhatched chickens were MG-positive, with the rate exceeding 90% in Breed 1. MG-positive unhatched chickens exhibited a significantly higher incidence of air sacculitis compared to healthy and weak chickens, characterized by cloudy, thickened air sacs with yellow cheese-like exudates (Figure 2).

These findings indicate that MS infection is rare in chicken embryos and 1-day-old chickens, whereas MG infection is more common, particularly in unhatched chickens, and is associated with air sac contamination.

### 3.3 Detection of offspring from positive parents

Fertilized eggs were collected over a 12-week period from 20 hens inseminated twice weekly with semen pooled from five roosters. Both the hens and roosters were from breeds with high MS-positive rates (Table 1). The infection status of the parent chickens was monitored from 27 to 38 weeks of age. Samples from the trachea, lungs, kidneys, and liver of 1-day-old offspring were pooled and tested for MS using qPCR. As shown in Table 3, all offspring tested negative for MS, despite intermittent shedding of the pathogen by the parent birds during the monitoring period. Notably, the MS-positive rates in the parent birds gradually increased over time, likely due to the artificial insemination process.

**Table 3:** MS positive parent at different ages and their offspring chickens.

**Chickens**	**27W**	**28W**	**29W**	**30W**	**31W**	**32W**	**33W**	**34W**	**35W**	**36W**	**37W**	**38W**
Hens	40% (8/20)	30% (6/20)	40% (8/20)	80% (16/20)	75% (15/20)	30% (6/20)	80% (16/20)	45% (9/20)	45% (9/20)	75% (15/20)	75% (15/20)	95% (19/20)
Cocks	20% (1/5)	0% (0/5)	0% (0/5)	60% (3/5)	60% (3/5)	40% (2/5)	100% (5/5)	40% (2/5)	40% (2/5)	60% (3/5)	40% (2/5)	40% (2/5)
Offspring	0% (0/20)	0% (0/18)	0% (0/20)	0% (0/19)	0% (0/17)	0% (0/18)	0% (0/38)	0% (0/36)	0% (0/36)	0% (0/36)	0% (0/36)	0% (0/36)

### 3.4 Detection of offspring chicken raised in different environment

**Figure 3:**
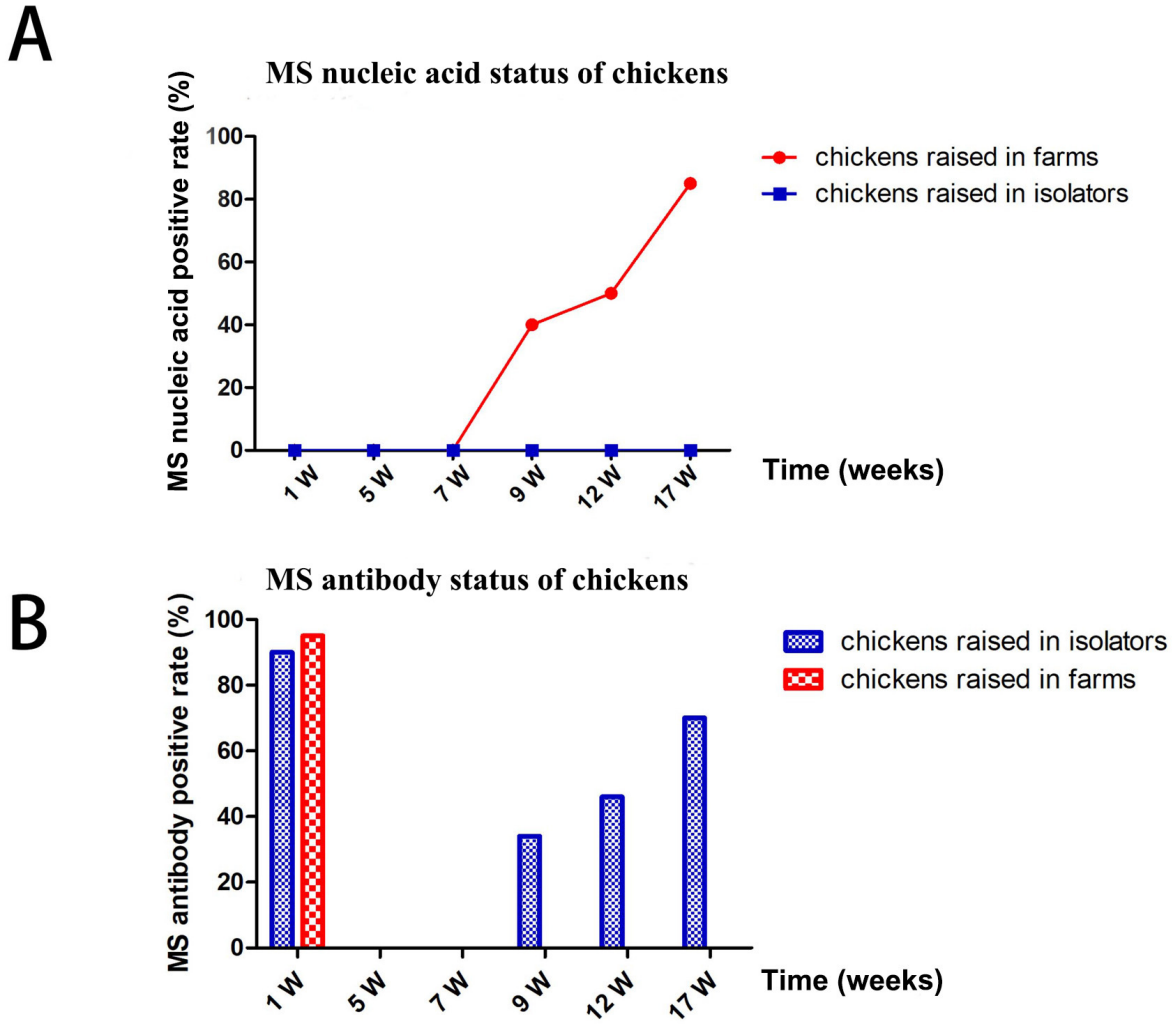
Differences in MS infection status of chickens fed in the isolators and in farms. **(A)** MS nucleic acid status of chickens. **(B)** MS antibody status of chickens.

Sixty 1-day-old chickens from farm A (with a high MS-positive rate) were randomly selected and raised in isolators (group 1) or breeding farms (group 2) for 17 weeks. MS DNA and antibodies were detected using qPCR and ELISA, respectively. Chickens in group 1 remained MS DNA-negative throughout the study. In contrast, chickens in group 2 tested positive for MS DNA starting at the 9th week, with the positive rate steadily increasing to 85% by the 17th week (Figure 3A).

MS antibodies were detected in over 90% of chickens in both groups at 1 week but became negative by the 7th week. Chickens in group 1 remained MS antibody-negative from 7 to 17 weeks. However, chickens in group 2 tested positive for MS antibodies at the 9th, 12th, and 17th weeks. The MS DNA-positive rates in group 2 were consistent with the MS antibody-positive rates (Figure 3B).

### 3.5 MLST typing of MS isolated from breeding chicken farms

A total of 29 *Mycoplasma synoviae* (MS) isolates were obtained from chickens with leg disease between 2019 and 2022 and subsequently sequenced. The DNA sequences were submitted to the PubMLST database for analysis. Among the 31 isolated MS strains, ST-34 (68.97%) and ST-93 (31.03%) were identified as the predominant strains, with ST-34 being the dominant strain (Table 4).

**Table 4:** MLST results of MS strains isolated from the farm.

**Separation time**	**Breed**	**Age (days)**	**Separation site**	**MLST result**
2022	Breed 1	49	Articular cavity	ST-93
2022	Breed 1	50	Palmula	ST-93
2022	Breed 3	112	Articular cavity	ST-93
2022	Breed 1	42	Joint fluid	ST-93
2022	Breed 1	36	Joint fluid	ST-34
2022	Breed 5	72	Choanal cleft swabs	ST-34
2022	Breed 4	75	Choanal cleft swabs	ST-34
2022	Breed 4	81	Choanal cleft swabs	ST-34
2022	Breed 5	80	choanal cleft swabs	ST-34
2022	Breed 2	55	joint fluid	ST-34
2022	Breed 1	38	choanal cleft swabs	ST-34
2022	Breed 2	55	Joint fluid	ST-34
2021	Breed 3	55	Joint fluid	ST-93
2021	Breed 3	55	Joint fluid	ST-93
2021	Breed 6	56	Joint fluid	ST-34
2021	Breed 2	46	Joint fluid	ST-34
2021	Breed 6	56	Joint fluid	ST-34
2021	Breed 2	61	Joint fluid	ST-34
2020	Breed 4	56	Lung, trachea	ST-34
2020	Breed 4	56	Lung, trachea	ST-34
2020	Breed 2	50	Joint fluid	ST-34
2020	Breed 2	62	Joint fluid	ST-34
2020	Breed 2	66	Joint fluid	ST-93
2019	Breed 5	50	Joint fluid	ST-93
2019	Breed 2	66	Joint fluid	ST-93
2019	Breed 5	57	Joint fluid	ST-34
2019	Breed 5	43	Swollen legs	ST-34
2019	Breed 1	50	Joint fluid	ST-34
2019	Breed 1	51	Joint fluid	ST-34

## 4 Discussion

*Mycoplasma synoviae* (MS) infections were first reported in the United States in the 1950s. Both the United States and the European Union have established large-scale monitoring and control programs, such as the National Poultry Improvement Plan (NPIP) and the European Poultry Breeding Control Plan, respectively (18). These programs involve extensive sampling at the end of the breeding period, regular testing, and culling of infected poultry flocks. Since 2010, MS infections have been increasingly reported in indigenous chicken populations in China (17). The unique characteristics of indigenous chicken breeds such as significant variability in breeding ages and management practices compared to white-feather broilers and layers pose challenges in disease prevention and control.

MS and MG infections in chickens can be transmitted both horizontally and vertically (19, 20). Horizontal transmission occurs through direct or indirect contact with contaminated personnel or aerosols, while vertical transmission primarily involves the transfer of pathogens via infected eggs from breeders to their offspring. Some studies (8, 19) have suggested that vertical transmission is considered the primary route of *Mycoplasma synoviae* (MS) infection, although the transmission rates through eggs vary depending on the strain and stage of infection. In Chinese indigenous chicken farming, practices such as multi-age mixed breeding and inadequate hygiene management may further facilitate the spread of *Mycoplasma* infections. (21). However, the specific dynamics of MS transmission under these conditions remain poorly understood.

In this study, distinct age-related patterns of MS infection were observed across the three farms. MS was not detected in 23 batches of chicken embryos, including yolk sacs and allantoic fluid, suggesting minimal infection levels in embryos. All flocks tested negative for MS up to 8 weeks of age, but positivity rates increased significantly in chickens older than 9 weeks. Those results indicated that vertical transmission at an early age is not the determinant cause of MS infection.

In monitoring 1-day-old chickens, we tracked offspring from two breeds, including healthy, weak, and unhatched chickens (due to incomplete pecking). A high incidence of air sacculitis was observed, characterized by thickened, cloudy air sacs, with severe cases exhibiting yellow, cheese-like exudates. qPCR results indicated low MS infection rates in these chickens, whereas MG positivity was significantly higher and strongly associated with air sacculitis. This suggests that vertical transmission of MG is more prominent, particularly in chickens with incomplete hatching.

Our previous studies have shown that MS vertical transmission is most likely during the first 4–6 weeks of the laying period. To investigate transmission dynamics, we isolated MS-positive breeders and monitored their offspring. While breeders maintained a low but persistent MS-positive status, their offspring remained MS-negative. However, MS infection rates of 1–3% were detected in certain breeds during the first 10 weeks of the laying period, indicating that horizontal transmission played a more significant role in these cases.

Environmental conditions also influenced MS infection rates. Chickens raised in isolators remained MS nucleic acid and antibody-negative up to 17 weeks of age, whereas those in breeder farms exhibited a 46.7% infection rate at 8 weeks and an 85% positivity rate by 17 weeks. These findings suggest that current vaccination and medication practices effectively block vertical transmission of MS, while horizontal transmission becomes a major risk around 7–8 weeks of age. According to our MLST typing results, the most prevalent MS strain was ST-34 (20/29). Those results are also correlated with other studies (6, 22). However, whether MLST subtype results are correlated with the horizontal and vertical transmission of MS needs further study.

Notably, although MS was not detected in isolator-raised chickens, some exhibited joint swelling. Pathogen analysis of joint fluid identified *Staphylococcus aureus* as the causative agent, and bacterial recovery experiments reproduced similar leg swelling (23–25). This suggests that other pathogenic infections, such as *S. aureus*, may contribute to clinical symptoms like leg swelling.

Antibiotics and vaccines remain the primary strategies for controlling MG and MS, though their effectiveness faces significant limitations. While antibiotics can temporarily reduce bacterial load and alleviate clinical symptoms, treatment discontinuation often leads to disease recurrence due to persistent colonization and potential resistance development. For MS control, the live attenuated MS-H vaccine can colonize the upper respiratory tract in negative flocks and may outcompete wild-type strains in low-exposure environments, but loses its protective advantage under high pathogen pressure. Similarly, MG vaccines like TS-11, 6/85 and F36 often fail to establish persistent colonization in endemic regions such as China, significantly compromising their protective efficacy against wild-type infections.

At the three local chicken breeding farms, both live and inactivated vaccines were routinely administered, complemented by frequent medication, to prevent outbreaks of MG and MS. Other studies have shown live vaccines (7, 26), inactivated vaccines and antimicrobial drugs (12, 27) significantly reduced the likelihood of MG/MS transmission. Both antibiotics and vaccines can control the horizonal transmission of MG and MS. And vaccines can stimulate maternal antibodies, further reduce the vertical transmission of MS. However, the differing vertical transmission rates between MG and MS may also stem from variations in the characteristics of their locally circulating strains. Given the widespread use of vaccines and antibiotics in China, circulating strains may have developed drug resistance and immune evasion mechanisms. To achieve complete disease eradication, it is imperative to implement real-time surveillance of prevalent strains and maintain regular updates of both vaccines and antimicrobial treatments.

## 5 Conclusions

This study provides a comprehensive analysis of *Mycoplasma synoviae* (MS) prevalence in Chinese indigenous chicken flocks and their offspring, with a particular focus on the vertical transmission dynamics of breeding flocks following immunization. The results indicate that the vertical transmission rate of MS is low, although it increases during the early egg production period, potentially elevating the risk of horizontal transmission under field conditions. In contrast, *Mycoplasma gallisepticum* (MG) demonstrates a higher vertical transmission rate across all ages and is frequently associated with air sacculitis.

## Data Availability

The datasets presented in this study can be found in online repositories. The names of the repository/repositories and accession number(s) can be found in the article/Supplementary material.

## References

[1] Yadav JP Tomar P Singh Y Khurana SK. Insights on *Mycoplasma gallisepticum* and *Mycoplasma synoviae* infection in poultry: a systematic review. Anim Biotechnol. (2021) 33:1711–20. 10.1080/10495398.2021.1908316 33840372

[2] Feberwee A de Wit S Dijkman R. Clinical expression, epidemiology, and monitoring of *Mycoplasma gallisepticum* and *Mycoplasma synoviae*: an update. Avian Pathol. (2021) 51:2–18. 10.1080/03079457.2021.1944605 34142880

[3] Landman WJM Feberwee A. Aerosol-induced *Mycoplasma synoviae* arthritis: the synergistic effect of infectious bronchitis virus infection. Avian Pathol. (2004) 33:591–8. 10.1080/03079450400013170 15763728

[4] Jeon E-O Kim J-N Lee H-R Koo B-S Min K-C Han M-S. Eggshell apex abnormalities associated with *Mycoplasma synoviae* infection in layers. J Vet Sci. (2014) 15:579–82. 10.4142/jvs.2014.15.4.579 24962418 PMC4269603

[5] Sun S-K Lin X Chen F Wang D-A Lu J-P Qin J-P. Epidemiological investigation of *Mycoplasma synoviae* in native chicken breeds in China. BMC Vet Res. (2017) 13:115. 10.1186/s12917-017-1029-0 28441945 PMC5405555

[6] Wei X Chen W Sun Q Zhong Q Yan Z Zhou Q. Epidemiological investigations and multilocus sequence typing of *Mycoplasma synoviae* isolates from chicken farms in China. Poult Sci. (2023) 102:102006. 10.1016/j.psj.2022.102006 37099877 PMC10165133

[7] Feberwee A Dijkman R Klinkenberg D Landman WJM. Quantification of the horizontal transmission of *Mycoplasma synoviae* in non-vaccinated and MS-H-vaccinated layers. Avian Pathol. (2017) 46:346–58. 10.1080/03079457.2017.1282602 28116916

[8] Ferguson-Noel N. Mycoplasmosis. In:Swayne DE Boulianne M Logue CM McDougald LR Nair V Suarez DL, editors. Diseases of Poultry. 14th ed. Vol 2. Hoboken, NJ: Wiley-Blackwell (2020). p. 907–25.

[9] Christensen NH Yavari CA McBain AJ Bradbury JM. Investigations into the survival of *Mycoplasma gallisepticum, Mycoplasma synoviae* and *Mycoplasma iowae* on materials found in the poultry house environment. Avian Pathol. (1994) 23:127–43. 10.1080/03079459408418980 18671077

[10] Marois C Picault J-P Kobisch M Kempf I. Experimental evidence of indirect transmission of *Mycoplasma synoviae*. Vet Res. (2005) 36:759–69. 10.1051/vetres:2005031 16120251

[11] Kleven SH. Control of avian *Mycoplasma i*nfections in commercial poultry. Avian Dis. (2008) 52:367–74. 10.1637/8323-041808-Review.1 18939621

[12] Garmyn A Vereecken M De Gussem K Depondt W Haesebrouck F Martel A. Efficacy of Tylosin and Tilmicosin Against Experimental *Mycoplasma gallisepticum* Infection in Chickens. Avian Dis. (2019) 63:359–65. 10.1637/11991-110818-Reg.1 31251538

[13] Gautier-Bouchardon AV. Antimicrobial Resistance in *Mycoplasma* spp. Microbiol Spectr. (2018) 6:10.1128/microbiolspec.ARBA-0030-2018. 10.1128/microbiolspec.ARBA-0030-2018 30003864 PMC11633602

[14] Gong X Chen Q Ferguson-Noel N Stipkovits L Szathmary S Liu Y Zheng F. Evaluation of protective efficacy of inactivated *Mycoplasma synoviae* vaccine with different adjuvants. Vet Immunol Immunopathol. (2020) 220:109995. 10.1016/j.vetimm.2019.109995 31877484

[15] Han S Wang Y Chang W Wang L Fang J Han J. Evaluation of the protective efficacy of six major immunogenic proteins of *Mycoplasma synoviae*. Front Vet Sci. (2024) 10:1334638. 10.3389/fvets.2023.1334638 38239753 PMC10794622

[16] Zhang G Han L Li Z Chen Y Li Q Wang S. Screening of immunogenic proteins and evaluation of vaccine candidates against *Mycoplasma synoviae*. NPJ Vaccines. (2023) 8:121. 10.1038/s41541-023-00721-y 37582795 PMC10427712

[17] Xue J Xu MY Ma ZJ Zhao J Jin N Zhang GZ. Serological investigation of *Mycoplasma synoviae* infection in China from 2010 to 2015. Poult Sci. (2017) 96:3109–12. 10.3382/ps/pex134 28637299

[18] Ewing ML Lauerman LH Kleven SH Brown MB. Evaluation of diagnostic procedures to detect *Mycoplasma synoviae* in commercial multiplier-breeder farms and commercial hatcheries in Florida. Avian Dis. (1996) 40:798–806. 10.2307/1592301 8980809

[19] Ter Veen C de Wit JJ Feberwee A. Relative contribution of vertical, within-farm and between-farm transmission of *Mycoplasma synoviae* in layer pullet flocks. Avian Pathol. (2020) 49:56–61. 10.1080/03079457.2019.1664725 31509002

[20] Mugunthan SP Kannan G Chandra HM Paital B. Infection, transmission, pathogenesis and vaccine development against *Mycoplasma gallisepticum*. Vaccines. (2023) 11:469. 10.3390/vaccines11020469 36851345 PMC9967393

[21] Sun S Lin X Liu J Tian Z Chen F Cao Y. Phylogenetic and pathogenic analysis of *Mycoplasma synoviae* isolated from native chicken breeds in China. Poult Sci. (2017) 96:2057–63. 10.3382/ps/pew484 28093481

[22] Zhang X Chen Y Xie D Guo M Ma S Chen M. Multi-locus sequence typing analysis of *Mycoplasma synoviae* isolates reveals unique sequence types in China. Vet Microbiol. (2021) 259:109101. 10.1016/j.vetmic.2021.109101 34166888

[23] Guo R Li Z Zhou X Huang C Hu Y Geng S. Induction of arthritis in chickens by infection with novel virulent *Salmonella pullorum* strains. Vet Microbiol. (2019) 228:165–72. 10.1016/j.vetmic.2018.11.032 30593363

[24] Li P Zhang M Hao G Sun S. Research note: hypervirulent arthritis-causing *Salmonella pullorum* isolated from Chinese native chicken breeds significantly decreased growth performance of chicks. Poult Sci. (2022) 101:101575. 10.1016/j.psj.2021.101575 34920386 PMC8686054

[25] Szafraniec GM Szeleszczuk P Dolka B. Review on skeletal disorders caused by Staphylococcus spp. in poultry. Vet Q. (2022) 42:21–40. 10.1080/01652176.2022.2033880 35076352 PMC8843168

[26] Ter Veen C Santman-Berends I Augustijn-Schretlen M Feberwee A. Quantification of the effect of vaccination on the control of horizontal transmission of *Mycoplasma synoviae* under field conditions. Avian Pathol. (2024) 13:1–7. 10.1080/03079457.2024.2358904 38771561

[27] Liu Y Dyall-Smith M Marenda M Hu H-W Browning G Billman-Jacobe H. Antibiotic resistance genes in antibiotic-free chicken farms. Antibiotics. (2020) 9:120. 10.3390/antibiotics9030120 32183177 PMC7148458

