# Supplementary material for: Transmission investigation of Mycoplasma synoviae in Chinese indigenous chickens
Source: Front Vet Sci. 2025 May 13;12:1555604. doi: 10.3389/fvets.2025.1555604 (PMC12108102; doi:10.3389/fvets.2025.1555604)
Supplement: Supplementary file 1 [file Table_1.docx]

**Table S1 The primers and probes for MS and MG**

| Primer and Probe | Sequences | Length |
| --- | --- | --- |
| MG-F | 5'-CTAGAGGGTTGGACAGTTATG-3’ | 139 bp |
| MG-R | 5'-GCTGCACTAAATGATACGTCAAA-3' |  |
| MG-P | 5'-(FAM)-CAGTCATTAACAACTTACCACCAGAATCTG-(MGB)-3' |  |
| MS-F | 5'-GAAGCAAAATAGTGATATCA-3' | 207 bp |
| MS-R | 5'-GTCGTCTCCGAAGTTAACAA-3’ |  |
| MS-P | 5'-(VIC)-AGCTACGCTACGGTGAATACGTTCTC-(TAMRA)-3' |  |
